# Inflammatory Processes Associated with Canine Intervertebral Disc Herniation

**DOI:** 10.3389/fimmu.2017.01681

**Published:** 2017-12-04

**Authors:** Marie Monchaux, Simone Forterre, David Spreng, Agnieszka Karol, Franck Forterre, Karin Wuertz-Kozak

**Affiliations:** ^1^Vetsuisse Faculty, Department of Clinical Veterinary Science, University of Bern, Bern, Switzerland; ^2^Competence Center of Applied Biotechnology and Molecular Medicine (CABMM), University of Zurich, Zurich, Switzerland; ^3^Vetsuisse Faculty, Institute of Veterinary Pathology, University of Zurich, Zurich, Switzerland; ^4^Department of Health Sciences and Technology, Institute for Biomechanics, ETH Zurich, Zurich, Switzerland; ^5^Schön Clinic Munich, Harlaching, Munich, Germany; ^6^Spine Research Institute, Paracelsus Medical University, Salzburg, Austria; ^7^Department of Health Sciences, University of Postdam, Postdam, Germany

**Keywords:** intervertebral disc herniation, inflammatory mediators, IL-1β, IL-6, IL-8, TNF-α, MAP kinase pathway, canine animal model

## Abstract

Intervertebral disc herniation (IVDH) is an important pathology in humans and also in dogs. While the molecular disease mechanisms are well investigated in humans, little is known about the inflammatory mediators in naturally occurring canine IVDH. The objective of this study was to investigate whether the involved proinflammatory cytokines in human IVDH are also key cytokines in canine IVDH and thus to elucidate the suitability of the dog as a model for human trials. 59 samples from 25 dogs with surgically confirmed thoracolumbar IVDH were collected and classified in three subgroups: herniated (H), affected non-herniated (NH) disc, and adjacent non-affected (NA) disc. Discs from 11 healthy dogs acted as controls (C). Samples were analyzed for IL-1, IL-6, IL-8, and TNF-α expression (qPCR/ELISA) as well as cell infiltration and activation of the MAP kinase pathways (immunohistochemistry). Gene and protein expression of all key cytokines could be detected in IVDH affected dogs. Canine IVDH was significantly associated with a higher gene expression of IL-6 (H > C, NH > C) and TNF-α (H > C, NH > C, NA > C) and a significant down-regulation of IL-1β (H < C). Dogs with spontaneous pain had significantly higher IL-6 mRNA compared to those with pain arising only upon palpation. An inter-donor comparison (H and HN relative to NA) revealed a significant increase of IL-6 gene expression (H > NA, NH > NA). IL-8 (H > C, NA > C) and TNF-α (NH > C) protein levels were significantly increased in diseased dogs while inversely, IL-6 protein levels were significantly higher in patients with better clinical outcome. Aside from resident IVD cells, mostly monocytes and macrophages were found in extruded material, with concomitant activation of extracellular signal-regulated kinase p38 in the majority of samples. Dogs with spontaneous IVDH might provide a useful model for human disc diseases. Although the expression of key cytokines found in human IVDH was also demonstrated in canine tissue, the inflammatory mechanisms accompanying canine IVDH diverges partially from humans, which will require further investigations in the future. In dogs, IL-6 seems to play an important pathological role and may represent a new potential therapeutic target for canine patients.

## Introduction

Intervertebral disc degeneration (IVDD) and intervertebral disc herniation (IVDH) are considered a major cause of acute and chronic low back pain, resulting in a tremendous economic burden worldwide (1–4). IVDD is characterized by progressive structural and functional changes, including a loss of hydrostatic properties of the normally highly hydrated nucleus pulposus (NP) (5), as well as increased vascular and neuronal ingrowth that is associated with chronic back pain (6). Recent human studies have identified proinflammatory cytokines, specifically IL-1β, IL-6, IL-8, and TNF-α, as pivotal contributors in the course of the pathophysiologic inflammatory cascade of intervertebral disc diseases and the development of neuropathic pain through irritation of ingrowing nerves (7–28). A herniated disc, presented as protrusion, extrusion, or sequestration, is a localized displacement of disc tissue in the epidural space (29), resulting in mechanical compression and chemical irritation of spinal nerves, which contribute to ischemia, radicular symptoms, and stimulation of the inflammatory cascade (30–39). Alongside of heredity (40, 41), multiple factors such as age and smoking are involved in the ethiopathogenesis of human IVDD and IVDH as well as in the initiation of an inflammatory cascade (42–45). Furthermore, obesity was highlighted as an important contributor to disc pathologies as it not only increases the load on the IVD (46–48), but also promotes inflammation *via* cytokine-release from adipocytes and recruited macrophages (49).

Measuring cytokine levels within the diseased tissue can provide a better understanding of the pathological process. As human tissue samples for research—especially as healthy controls—are scarce, more complex pathological investigations and testing of new therapeutic approaches often require animal experiments (50). Currently established models are predominantly based on artificially induced disc pathology through mostly invasive manipulations (e.g., stab incision in rodents), which lack similarities to the human pathology (51). In contrast, animal models based on spontaneously occurring disc pathologies, such as canine IVDH, share important similarities to the clinical presentation, pathology, lesion morphology, diagnostic, treatment, and recovery with human IVDH (50, 52, 53). IVDH in dogs has an incidence of 2% of all admissions in referral clinics and occurs predominantly in chondrodystrophic breeds, such as Beagle, Dachshund, Shi-Tzu, and French Bulldog (54–59). Similar to humans, the prevalence of overweight and obesity in dogs is increasing, being as high as 34% in the US and 25% in the UK (60, 61), with a higher risk factor for disc extrusion in dogs with higher body score index (62). Despite the potential relevance of canine IVDH as a human disease model, little in depth research has been conducted to determine its pathological processes in dogs, specifically regarding the role of inflammatory mediators in disease progression and pain development. Thus far, existing data indicate that in the early phase of canine IVDH, mRNA concentration of IL-6, a possible promoter of inflammation and apoptosis of resident glial cells, was significantly upregulated, whereas returning to baseline values in later stages of the disease. In comparison, mRNA concentration of IL-8, a potent chemokine and early mediator of inflammation, was strongly upregulated in the acute and subacute onset of IVDH. A trend of higher TNF-α mRNA concentration in acute IVDH could also be shown (63). Controversially, Karli et al. demonstrated a downregulation of IL-1β, IL-6, and TNF-α mRNA concentrations over the whole course of canine IVDH, but highlighted an upregulation of IL-8 mRNA concentration in the acute stage of the disease, which decreased when treated with non-steroidal anti-inflammatory drugs (64).

Based on the conflicting data found in the current literature, the aim of this study was to identify whether the human key cytokines are expressed in canine IVDs and to determine pathological alterations in their expression by comparing normal IVDs to diseased IVDs with IVDH, with the overall goal to provide further information on the validity of the canine disc disease model. Concomitantly, we also assessed neurologic function, pain status, and clinical outcome in affected dogs.

## Materials and Methods

### Patients, Controls, and Tissue Samples

Canine disc material was collected from dogs with confirmed thoracolumbar IVDH during decompressive surgical procedures at our referral institution (November 2013 to February 2015). All samples were collected from actual referred patients and not from laboratory animals. Herniated (H) and non-herniated (NH) IVD samples were both collected from the IVDH: affected level, with H = prolapsed part and NH = contained part. Non-affected (NA) IVDs were collected from an adjacent spinal level (prophylactic disc fenestration). Inclusion criteria for IVDH patients: well-documented records of onset of clinical signs and pretreatment, neurologic findings, diagnosis, course of the disease, ethical approval, and signed owner consent.

In addition, healthy control IVDs (C) were collected from dogs included in an unrelated animal study or patients without any history or signs of spinal disease and without any anti-inflammatory pretreatment euthanized for other health reasons. These samples were directly surgically collected after euthanasia within 15 min to avoid potential artifacts in measurements of the cytokines (65–67).

In the patient group, breed, age, gender, duration of neurologic signs, pretreatment, neurologic grade, severity of pain, localization of IVD extrusion, and outcome were recorded. Duration of neurologic signs were defined as the time between the onset of clinical signs and surgery and was grouped as follows: decompression within the first 24 h after onset of clinical signs (acute), between 24 h and 7 days of onset (subacute), or after more than 7 days (chronic) (68, 69). A complete neurological examination was performed at the time of admission, maximally 12 h before surgery, at the time at discharge, and at a follow up examination between 2 and 4 weeks after discharge. The neurologic condition and pain were graded as shown in Table 1 (64, 70, 71).

**Table 1:** Clinical scoring system.

Grade	Neurological condition	Pain
Zero		Neither spontaneous pain (vocalization, reluctance to move) nor pain on palpation of the spine
One	Spinal hyperesthesia only	No spontaneous pain, but discomfort (licking, withdrawal) on palpation of the spine
Two	Ambulatory paraparesis	Spontaneous pain and excessive pain (vocalization, aggressive behavior) on palpation of the spine
Three	Non-ambulatory paraparesis	
Four	Paraplegia with intact nociception	
Five	Paraplegia without nociception	

### Diagnostic Imaging and Surgical Treatment

Anesthesia and analgesia were conducted under a standard clinical protocol for each patient (44). Magnetic resonance imaging (MRI) of the thoracolumbar spine was performed (Philips Panorama HFO, 1.0-T open system, Philips Medical Systems Nederland B.V., The Netherlands) to define the IVD extrusion site. Immediately after MRI, patients underwent surgery for discogenic back pain and to decompress the spinal cord. A standard hemilaminectomy at the site of extrusion, fenestration of the affected and one of the adjacent discs (cranial or caudal) were performed, allowing to take three samples of disc material (H, NH, and NA). For cytokine identification, removed disc material was collected under sterile conditions, snap frozen directly within 15 s in liquid nitrogen and stored at −80°C until sample preparation. For immunohistochemical and histological analysis, the tissue was fixed in 10% buffered formalin (1–5 days), embedded in paraffin, and cut into 5 µm sections.

### Real-time PCR (RT-PCR)

For RNA isolation, each frozen disc sample was transferred into a precooled custom-made mortar, filled with liquid nitrogen and manually grinded under RNAse free conditions. The frozen tissue powder was transferred into 500 µl Trizol (Life Technologies, Van Alben Way, CA, USA), homogenized three times for 30 s (Polytron PT 2500 E, Kinematica, Luzern, Switzerland), incubated for 5 min at RT and centrifuged at 12,000 *g* for 10 min at 4°C.

The supernatant was mixed with 250 µl chloroform, vortexed for 30 s, incubated at RT for 15 min, centrifuged at 12,000 *g* for 15 min at 4°C and the aqueous phase recovered. RNA was precipitated by adding 125 µl isopropanol and 125 µl of high salt precipitation solution (0.8 M sodium citrate, 1.2 M sodium chloride), washed with 500 µl of 75% ethanol and dissolved in 25 µl RNase free water after complete removal of ethanol. The quantity of the isolated RNA was determined spectroscopically (NanoDrop^®^ Lite, Thermo Fisher Scientific, Wilmington, DE, USA) and the quality confirmed by the sample’s OD 260/280 (1.8–2.0; samples with lower quality were excluded from further analyses). cDNA was prepared using the TaqMan Reverse Transcription kit (TaqMan^®^ Reverse Transcription Reagent, Life Technologies, Van Alben Way, CA, USA) according to the manufacturer’s instruction. RT-qPCR was conducted on the CFX96 Touch qPCR Machine (Bio-Rad Laboratories, Hercules, CA, USA) as previously described (46, 47), by mixing 5 µl of TaqMan^®^ Fast Advanced Master Mix (Life Technologies, Van Alben Way, CA, USA) with 10 ng cDNA in RNAse free water (4.5 µl in total) and 0.5 µl of canine TaqMan primers/probes (TaqMan^®^ Gene Expression Assays, Life Technologies, Van Alben Way, CA, USA) (Table 2).

**Table 2:** TaqMan primers/probes and sequence accession number.

Target gene	Assay number	Accession number
IL-1β	Cf02671951_g1	NM_001037971.1
IL-6	Cf02624282_m1	NM_001003301.1
IL-8	Cf02624283_m1	NM_001003200.1
TNF-α	Cf02624261_m1	NM_001003244.4
TATA box-binding protein (TBP)	Cf02637232_m1	XM_005627735.2

Each sample was run in duplicate for each gene, the CT value means were calculated and the comparative CT method was used for data evaluation [relative quantification (RQ)]. Samples with gene expression lower than the detection limit were attributed an artificial maximal CT value of 43, using the selection of the median as aggregation method between samples with the same experimental condition to avoid statistical and mathematical issues (72).

For group comparisons between H, NH, NA, and C, results were calculated as 2^−ΔCT^ values, i.e., the expression of each target gene was normalized relative to the expression of the house keeping gene [canine TATA box-binding protein (TBP)], using the classical ΔCT method and the following algorithm:

$${\text{RQ = 2}}^{-\text{ (Ct}{\left(\text{H}\right)}_{\text{TargetGene}}\;-\text{ Ct}{\left(\text{H}\right)}_{\text{TBP}}\text{)}}(\text{identically for NH, NA, C}).$$

For interdonor calculations, an additional normalization step (ΔΔCT) was performed (ΔCT of H or NH minus ΔCT of NA, of the same animal). Results were calculated as 2^−ΔΔCT^ values and shown as fold change in expression between affected discs (H, NH) and internal control discs (NA). The following algorithm was used:

$${\text{RQ = 2}}^{-\text{[(Ct}{\left(\text{H}\right)}_{\text{TargetGene}}-\text{Ct}{\left(\text{H}\right)}_{\text{TBP}}\text{) }-\text{(Ct}{\left(\text{NA}\right)}_{\text{TargetGene}}-\text{Ct}{\left(\text{NA}\right)}_{\text{TBP}}\text{)]}}\;(\text{identically for NH}).$$

The intra-assay CV was 0.74% (*n* = 131).

### Total Protein and ELISA

ELISA samples were weighed and pulverized as described above, but with immersion in T-PER reagent (Tissue Protein Extraction Reagent, Thermo Fisher Scientific, Wilmington, DE, USA) at a ratio of 100 µl T-PER reagent per 10 mg tissue. The lysates were homogenized and purified following a standardized protocol. Briefly, samples were centrifuged at 12,000 *g* at 4°C for 10 min, the supernatant was collected and total protein was determined by the Bradford assay (Bio-Rad Protein Assay Dye Reagent Concentrate, Bio-Rad Laboratories, Hercules, CA, USA), with absorbance measurement at 585 nm (Infinite^®^ 200 Pro, Tecan Group Ltd., Männedorf, Switzerland). The protein levels of IL-1β, IL-6, IL-8, and TNF-α were detected with canine specific ELISAs (MyBioSource, San Diego, CA, USA for IL-1β, IL-6, IL-8, and Quantikine ELISA Kit, R&D Systems Inc., Minneapolis, MN, USA, for TNF-α) according to the manufacturer’s instructions, with a sensitivity of 1 pg/ml for IL-1β, IL-6, and IL-8 kit and 4.2 pg/ml for TNF-α. Sample concentration was calculated based on a standard curve and normalized to total protein content. Results are demonstrated either as group comparisons (H, NH, NA, C: cytokine content/total protein in pg/mg) or as interdonor comparisons (H and NH relative to NA: fold change). The median intra-assay CV is 9.0% (range 5.3–15%) and the median interassay CV 10.8% (range 9.3–15%), as provided by the supplier.

### Immunohistochemistry

Serial sections were deparaffinized, heat-mediated antigen retrieval was conducted (95°C, 20 min) and sections were incubated with primary antibodies (RT, overnight) identifying infiltrating cells (polymorphonuclear, mononuclear, macrophages, multinucleated giant cells) and MAP kinase (MAPK) pathway activation (Table 3). Subsequently, sections were treated with an appropriate primary antibody (Marker) and washed 3× with Tris buffer before the secondary antimouse IgG was applied (1:200 dilution, 30 min). The amino-9-ethyl-carbazole substrate kit (Dako) was employed as a chromogen. Finally, the sections were counter-stained with Gill’s hematoxylin for 3 min, and cover-slipped with an aqueous mounting media (Glicerine, Sigma-Aldrich).

**Table 3:** Detailed antibody information.

Marker	Provider	Order nr.	Dilution	Read-out
CD18	P. Moore	CA16.3C10	1:10	Cell infiltration
CD3	Dako	A0452	1:600	
vWF8	Dako	M0616	1:400	
p-JNK	Cell Signaling Technologies	PA5-21343	1:50	MAP kinase activation
p-p38	Cell Signaling Technologies	ab32142	1:50	
p-ERK1/2	Cell Signaling Technologies	MAI-13041	1:50	

All slides were examined and semiquantitatively evaluated independently by two investigators for infiltrating cell types in the extruded NP as well as activation of the MAPK pathway [c-Jun NH2-terminal kinase (JNK), p38 isoforms of MAPK (p-38), extracellular signal-regulated kinases (ERKs)]. The following grades were used: (−) negative; (±) weakly (less than 5% of cells positive); (+) moderately (5–25% of cells positive); (++) strongly (more than 25% of cells positive).

### Statistical Analysis

For all results, an outlier calculation was performed and outliers removed before statistical analysis (MedCalc 16.8/2016, http://www.medcalc.org). Summary statistics were performed and normality assessed using D’Agostino-Pearson tests. As data were versatile regarding its distribution, nonparametric statistics were additionally used except for the interdonor comparison. A Kruskal–Wallis test was used to identify any association between gene and protein expression of cytokines with location of IVDH, duration of clinical signs, neurologic grade, pretreatment and outcome. Differences in gene or protein expression of cytokines between the samples of affected and control animals were evaluated using a Mann–Whitney *U*-test. The subgroup comparison was carried out stepwise by a Wilcoxon signed-rank test. The Bonferoni *p*-value adjustment was used to control for multiple comparisons. For inter-donor comparisons of gene and protein expression, an independent sample *t*-test and a Mann–Whitney *U*-test was performed, respectively. A Spearman’s rank correlation was used to examine the relationship between gene expression and protein level. Threshold value for statistical significance was set up to *p* < 0.05 and data are presented as boxplots, with median and 95% confidential interval (CI) unless otherwise stated.

## Results

### Clinical Data

Twenty-five dogs with surgically confirmed thoracolumbar IVDH were included in the study. All canine patients (25 of 25 cases) were small and medium-breed dogs (<20 kg) with a median body weight at admission of 9.0 kg (range 3.6–19.0 kg). The dogs were classified as chondrodystrophic breeds with the Dachshund (*n* = 5), Cocker Spaniel, Coton de Tuléar and the French Bulldog (each *n* = 2) and the Bichon Frisé, Cavalier King Charles Spaniel, Jack Russel Terrier, Pekingese, Miniature Poodle, Pug Dog, Shi-Tzu, and the Yorkshire Terrier (each *n* = 1) or as non-chondrodystrophic or mixed breeds with the Border Collie (*n* = 1) and the Mixed Breed Dog (*n* = 5) (57, 73, 74). The median age was 5 years (range 3–13 years). Sixteen dogs were male intact, four were female intact, three were female spayed, and two were male castrated. The median duration of clinical signs until surgery was 3 days (range 1–365 days). When classified into three groups, there were 3 acute, 14 subacute, and 8 chronic cases. Ten dogs were referred without pretreatment, five had been pretreated with steroids, seven with NSAIDs, and three with a combination of both, pooled together as pretreated dogs. The neurologic status before surgery was Grade 2 in 11, Grade 3 in 6, Grade 4 in 5, and Grade 5 in 5 dogs. Pain score revealed 2 dogs with Grade 0, 14 dogs with Grade 1, and 9 animals with Grade 2. The spinal level of IVDH in the affected dogs were L2–L3 in eight dogs, Th12–Th13 in six dogs, Th11-Th12, L1–L2, L3–L4 in each three dogs, and Th10–Th11, Th13–L1 in each one dog. Fifteen dogs had a good outcome, six showed an improvement, but were not ambulatory, and four showed no improvement within 1 month after surgery. In the latter case, two of them were euthanized because of poor prognosis, a follow-up call unveiled that the other two dogs are still in physiotherapy treatment showing no improvement in the last 7 and 9 months.

Eleven euthanized dogs without signs of IVDH were included as control group. The median age of the control group was 4.5 years (2–11 years). Ten dogs were female spayed and one dog was female intact. There were 10 beagles and 1 was a mixed breed dog.

### Diagnostic Imaging and Surgical Treatment

All 25 affected dogs underwent surgical decompression of the spinal cord immediately after diagnostic imaging, including the collection of the samples. Three different samples (H, NH, and NA) were collected in 14 dogs, two different samples (H, NH) in 6 dogs, and one sample (H) in 5 dogs, resulting in a total of 59 samples. The median sample weight was 30.62 mg (range 1.7–112.3 mg). Six disc samples were excluded: four NHs and one H were excluded due to very small size and one NH sample demonstrated inconsistent housekeeping gene expression and out of range protein values. From the remaining 53 samples, 11 tissue samples weighting more than 50 mg were equally divided in samples for PCR and ELISA testing. 18 smaller samples were solely assigned to PCR testing and 24 smaller samples solely to ELISA testing. In the C group, we collected 11 samples in 11 dogs in the Th13–L1–L2 IVD space, with a median sample weight of 60.87 mg (range 30.3–133 mg). One dog was completely omitted because of RNA impurity. From the 10 remaining control samples, 4 samples weighting more than 50 mg were equally split into samples for PCR and ELISA. Three samples each were solely assigned to PCR and ELISA testing.

### Cytokine Detectability

We could demonstrate gene and protein expression of the selected proinflammatory cytokines IL-1β, IL-6, IL-8, and TNF-α in IVDH in dogs.

### Gene Expression

**Figure 1:**
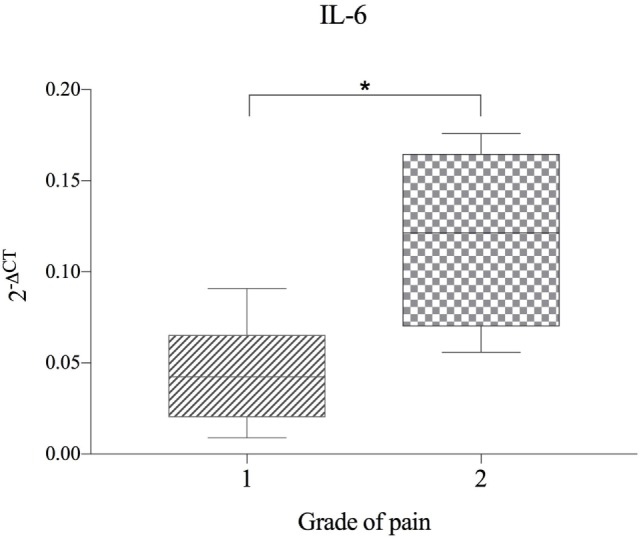
Gene expression of IL-6 in herniated disc material, compared between dogs with differing grade of pain (*n* = 11) and shown as 2^−ΔCT^. Grade 1 = no spontaneous pain, but discomfort upon palpation of the spine. Grade 2 = spontaneous pain and excessive pain upon palpation of the spine. Asterisks indicate statistical significance between groups at *p* < 0.05 (*).

**Figure 2:**
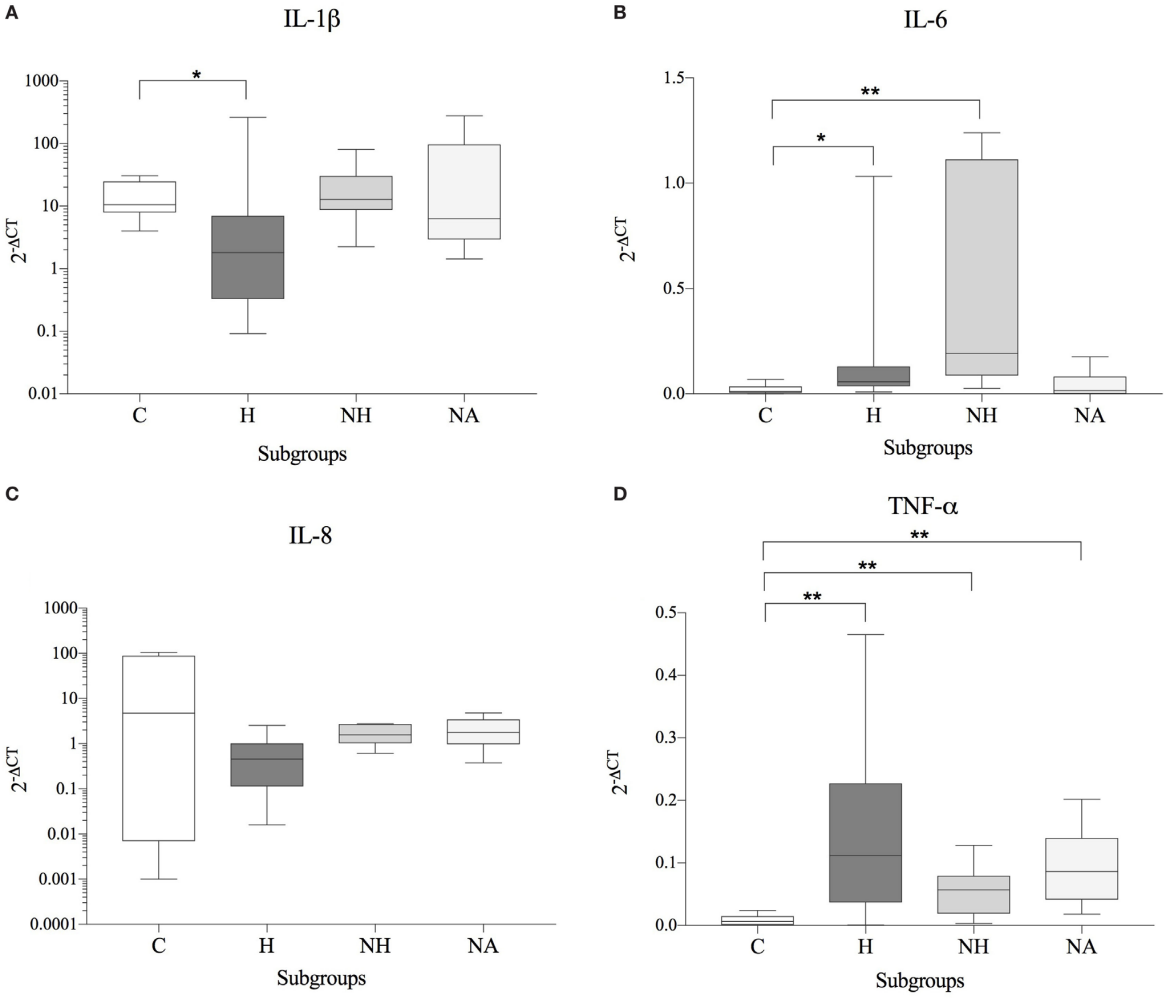
Gene expression data (subgroup comparison, independent samples, *n* ≥ 6 per subgroup) of **(A)** IL-1β, **(B)** IL-6, **(C)** IL-8, and **(D)** TNF-α, compared between herniated (H), non-herniated (NH), non-affected (NA) as well as healthy control (C) intervertebral disc material. Data are shown as 2^−ΔCT^. Asterisks indicate statistical significance between groups at *p* < 0.05 (*) and *p* < 0.01 (**).

No significant correlation between IVDH location, duration of clinical signs or outcome, and the genes of interest was demonstrated, but the gene expression of IL-6 was significantly higher (*p* = *0.043*) in pain grade 2 compared to grade 1 (Figure 1). Pooled results of all dogs in the respective groups (C, H, NH, NA) showed a significant downregulation of IL-1β in H compared to C, but no difference between NH or NA and C was found. IL-6 expression was significantly higher in H and NH compared to C, but not in NA. TNF-α expression showed significantly higher levels in H, NA, and NH compared to C. Gene expression of IL-8 showed no significant difference among the groups. All results are shown in Table 4 and Figure 2.

**Table 4:** Statistical results of gene expression data (subgroup comparison, independent samples).

Cytokine	Control IVD	Median (95% CI)	Comparative IVD	Median (95% CI)	*p*-Value
IL-1β	C	10.556 (5.927–27.650)	H	1.798 (0.329–7.156)	0.018
IL-6	C	0.012 (0.004–0.052)	H	0.057 (0.034–0.139)	0.013
			NH	0.178 (0.045–1.114)	0.006
TNF-α	C	0.007 (0.001–0.022)	H	0.112 (0.036–0.230)	0.008
			NA	0.087 (0.024–0.186)	0.008
			NH	0.060 (0.019–0.080)	0.007

**Figure 3:**
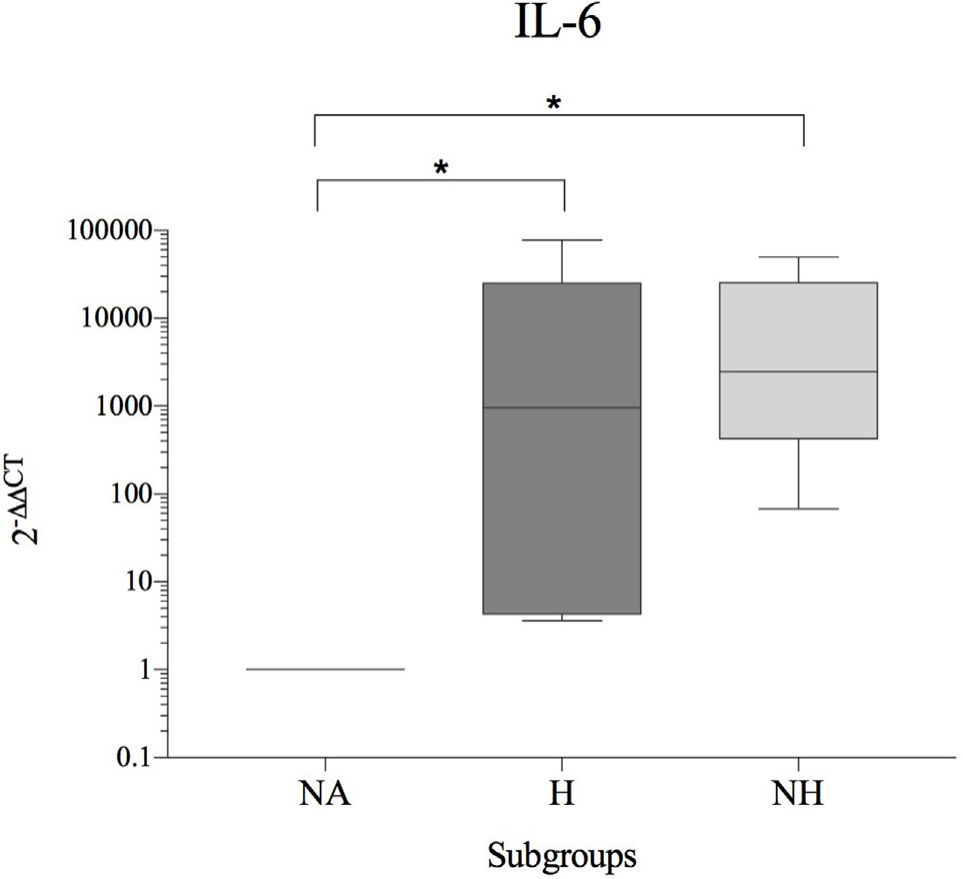
Fold change in IL-6 gene expression (interdonor comparison, *n* = 6 per subgroup) between each dog’s non-affected IVD material (NA) and the same dog’s herniated (H) or non-herniated (NH) intervertebral disc material. Data are shown as 2^−ΔΔCT^. Asterisks indicate statistical significance between groups at *p* < 0.05 (*).

As patients demonstrated differences in their basal cytokine levels, hence resulting in high donor–donor variations in subgroup comparisons, an interdonor comparison was conducted (H and HN relative to NA = internal control for each patient). A significant increase of IL-6 was observed for H and NH compared to NA. In contrast, gene expression of IL-1β, IL-8, and TNF-α were either unchanged or reduced in diseased compared to NA IVDs. All results are shown in Table 5 and Figure 3.

**Table 5:** Statistical results of interdonor gene expression data (subgroup comparison, dependent samples).

Cytokine	Control IVD	Comparative IVD	Median (95% CI)	*p*-Value
IL-6	NA	H	498.625 (3.722–20,901.118)	0.031
		NH	2,473.292 (158.129–43,724.429)	0.031

### Protein Expression

**Figure 4:**
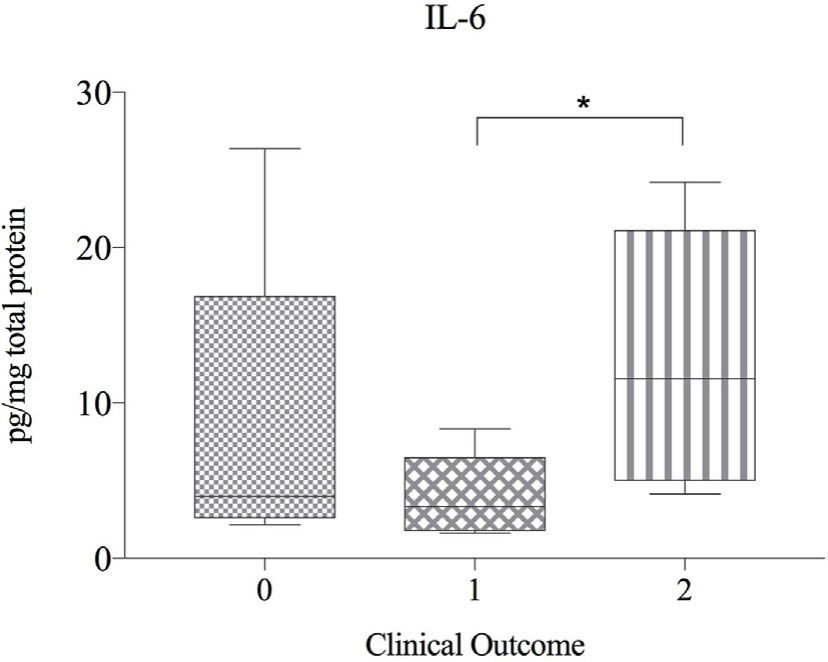
Protein expression of IL-6 in herniated disc material, compared betwen dogs with differing clinical outcome (*n* = 20) and shown as picogram of IL-6 per milligram total protein. Grade 0 = lack of improvement. Grade 1 = improvement of neurologic status, but not able to walk. Grade 2 = recovery of ambulation or at least one grade in neurologic grades 1 and 2. Asterisks indicate statistical significance between groups at *p* < 0.05 (*).

There were no significant correlations between cytokine protein levels and IVDH location, severity of pain, pretreatment or outcome and concentration of cytokines, but IL-6 was found to be significant higher in the clinical outcome group 2 compared to group 1 (Figure 4).

**Figure 5:**
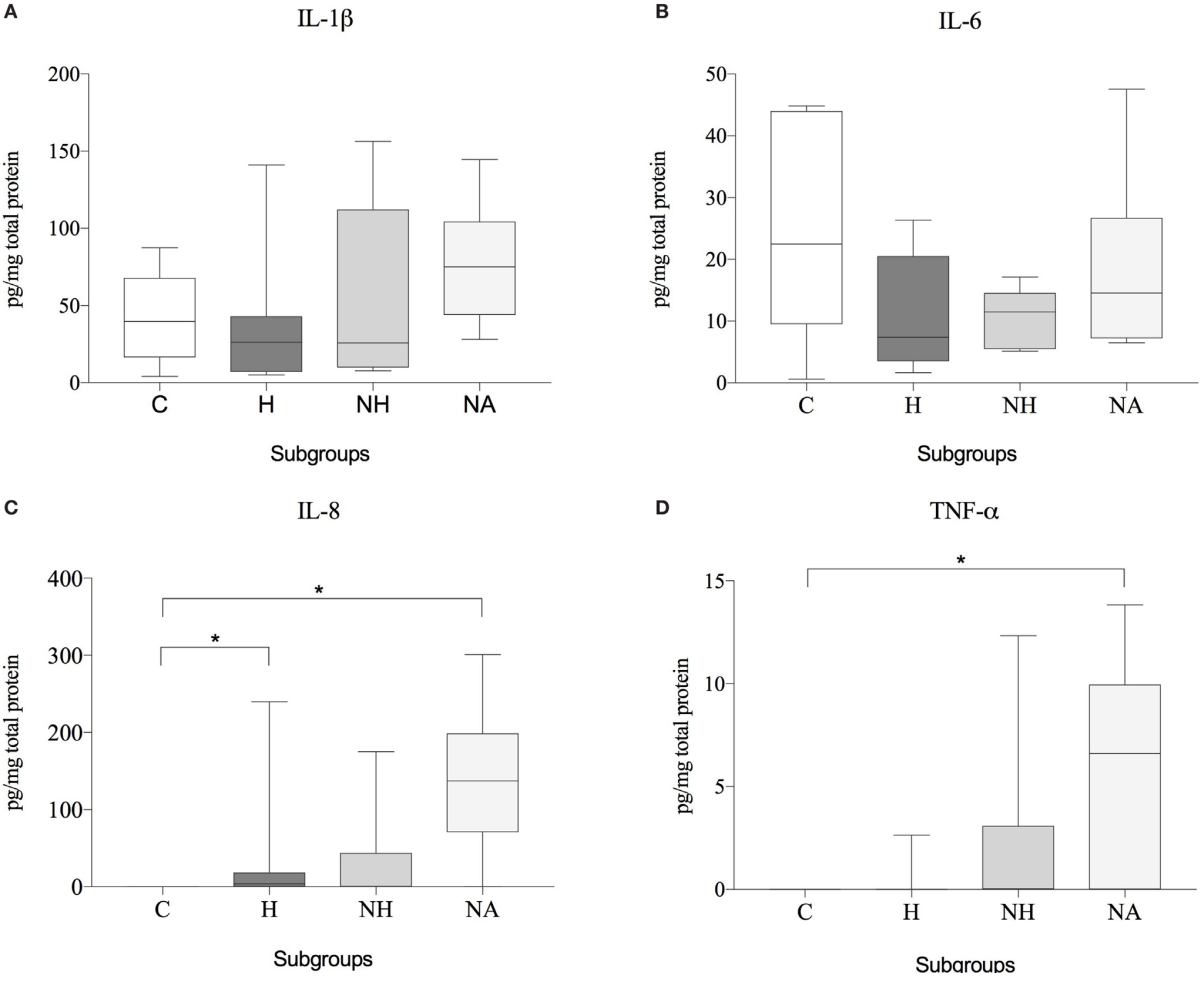
Protein expression data (subgroup comparison, independent samples, *n* ≥ 6 per subgroup) of **(A)** IL-1β, **(B)** IL-6, **(C)** IL-8, and **(D)** TNF-α, compared between herniated (H), non-herniated (NH), non-affected (NA) as well as healthy control (C) intervertebral disc material. Data are shown as picogram of each cytokine per milligram total protein. Asterisks indicate statistical significance between groups at *p* < 0.05 (*).

No significant differences of IL-1β or IL-6 protein expression were observed between the various groups. The concentration of IL-8 was significantly higher in H and NA compared to C, but no difference was found between NH and C. TNF-α showed significantly higher protein levels in NH compared to C, but not for H or NA. Results are shown in Table 6 and Figure 5.

**Table 6:** Statistical results of protein expression data (subgroup comparison, independent samples).

Cytokine	Control IVD	Median (95% CI)	Comparative IVD	Median (95% CI)	*p*-Value
IL-8	C	0.00001 (0.00001–0.00001)	H	3.925 (0.00001–17.823)	0.012
			NA sufficient	126.294 (13.728–187.946)	0.035
TNF-α	C	0.00001 (0.00001–0.00001)	NH sufficient	0.00001 (0.00001–9.924)	0.042

Like for gene expression, an additional inter-donor comparison was conducted, with NA as the internal control. However, no statistical differences were found between diseased (H, NH) and NA IVDs due to a level of variability and low sample numbers (data not shown).

No significant relationship was found between gene expression and corresponding protein concentration (IL-1β *r*s = 0.02; 95% CI, −0.66–0.67, *p* = 0.97; *IL-6 r*s = 0.30; 95% CI, −0.41–0.78; *p* = 0.41; IL-8 *r*s = −0.25, 95% CI, −0.85–0.62, *p* = 0.59; TNF-α *r*s = −0.51, 95% CI, −0.86–0.18, *p* = 0.13). Interestingly, a significant association in gene expression between IL-6 and IL-8 (*r*s = 0.79, 95% CI 0.08–0.97, *p* = 0.036) was found in the control group. In herniated samples, IL-1β protein correlated with IL-6 (*r*s = 0.71; 95% CI, 0.36–0.88, *p* = 0.001), IL-8 (*r*s = 0.54; 95% CI, 0.09–0.80, *p* = 0.02) as well as TNF-α concentrations (*r*s = 0.55; 95% CI, 0.11–0.81, *p* = 0.02) measured by ELISA.

### Immunohistochemistry

**Figure 6:**
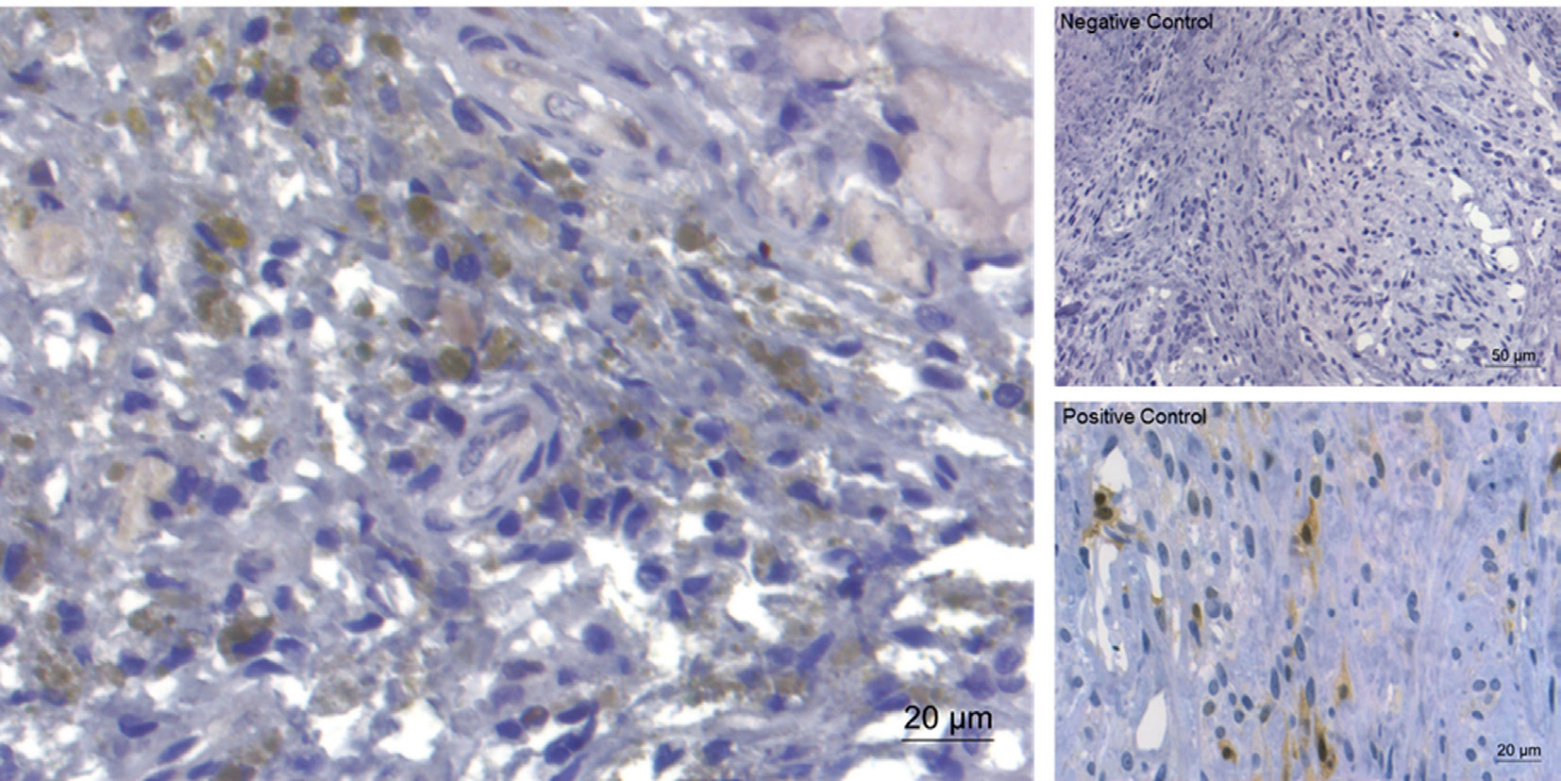
Immunohistochemical staining for extracellular signal-regulated kinase (ERK)1/2 of extruded canine intervertebral disc material (left). Expression of ERK (brownish coloration) was detected in the majority of the examined slides. On the right, negative control and positive control (canine mammar carcinoma) are depicted.

Monocytes and macrophages were the most infiltrating cell population encountered in canine herniated IVD material. Sporadically, giant cells resembling notochordal cells were observed. CD 18 was highly expressed (++) in all samples, whereas detection of CD3 and vWF8 detection was semi-intractable and error-prone, hence not being shown. Expression of activated (phosphorylated) ERK (++) (Figure 6) and p38 (+/++) could be detected in most of the examined slides, whereas pJNK (−/+) was only sporadically detectable (not shown).

## Discussion

Previous research on human IVD tissue has demonstrated that degeneration and herniation coincides with increased levels of inflammatory mediators, such as IL-1β, IL-6, IL-8, and TNF-α (8). In the present study, a comparison between diseased (herniated IVDs = H; NH) and control (C) IVDs of dogs demonstrated that disc disease was associated with significantly higher gene expression of IL-6 (H vs. C, NH vs. C) and TNF-α (H vs. C, NH vs. C). On the protein level, a significant increase of IL-8 and TNF-α in IVDs of diseased dogs compared to controls was found. A patient-specific comparison between the diseased (H, NH) and the IVDs excised from an adjacent level (NA) of the same dog revealed a pronounced pathological elevation of IL-6 (H vs. NA, NH vs. NA). In contrast, mRNA levels of IL-1β, IL-8, and TNF-α were either unchanged or reduced in diseased compared to NA IVDs of patients.

Previous studies on canine disc disease found IL-1β to be either undetectable or downregulated in dogs, which contrasts human disc pathology (64, 75). This was confirmed by our findings, showing a significant downregulation of the gene expression of IL-1β in herniated disc material (H) compared to controls, a downregulation in the affected disc material (H, NH) of the paired samples and no significant findings on the protein level.

In human IVDH, higher protein levels of the proinflammatory cytokine IL-6 were detected in different studies (10, 23, 25, 76), hence being discussed as a prognostic factor and therapeutic target. Thus far, results on IL-6 expression during canine disc disease are contradictory (63, 64, 75, 77). According to our findings, IL-6 seems to have a pivotal role, with its gene expression being significantly upregulated in the affected disc material (H, NH) of the independent and paired samples, unrelated to the duration of the clinical signs. Interestingly, we noticed a correlation between IL-6 mRNA levels and the severity of pain, with mRNA expression being significantly higher in those canine patients with basal pain than in those with pain arising only upon palpation. To the authors’ knowledge, this has not been described before in canine IVDH, but might be in accordance with recent research in humans that has increasingly pointed toward IL-6 being a crucial factor in pain development (78, 79). However, IL-6 is not only a proinflammatory cytokine with significance in inflammation and diseases, but is also known to have regenerative or anti-inflammatory properties (80). Our results indicated that canine patients showing higher concentration of IL-6 protein levels in their IVD had a better outcome and were more likely to regain ambulation. This contrasting finding might be explained by the dual function of IL-6, suggesting that with time, IL-6 might control inflammation by acting as an anti-inflammatory cytokine (81, 82). IL-6 originates directly from IVD cells (76) and also from T-cells and macrophages (83–86), the latter constituting the main cell population of the inflammatory infiltrates in the epidural space after IVD extrusion in our samples. Based on our findings, IL-6 could potentially have also a beneficial role in the late onset of the inflammatory cascade of canine IVDH. Therefore, therapeutic blockage for IL-6 should be carefully considered.

In this study, we also analyzed the expression of IL-8, which has previously been shown to be strongly upregulated in the acute phase of canine IVDH (63, 64, 77), yet with a negative correlation between its expression and the duration of spinal cord injury secondary to IVDH (77). As IL-8 is thus thought to be an early disease marker with time-dependent expression and as our patients were mostly seen for treatment at a late stage of the disease, it explains why we could not demonstrate a significant upregulation of IL-8 mRNA, but of IL-8 protein in diseased discs. Interestingly, a significant upregulation of the protein level of IL-8 in independent samples could as also be demonstrated in NA disc material, suggesting a possible secretion of IL-8 in canine disc material before the effective herniation of the affected IVD and hence further supporting a possible early stage inflammation as a trigger for IVDH.

Together with IL-1β, TNF-α is likely the most studied cytokine in human IVDD (11, 17, 28, 87, 88), inducing matrix degrading enzyme expression and upregulation of nerve growth factor. TNF-α is hence suggested to be have an important role in the development of hyperalgesia and chronic pain after IVDH in humans (7, 16, 28, 89–92). Contradictory results have been found on TNF-α expression in canine IVDH, ranging from non-detectability (64, 75, 77) to disease-related elevation (albeit non-significant) (63). In accordance with Spitzbart et al. (63), as well as with human studies, we could demonstrate a significant upregulation of TNF-α on the gene expression level (H, NH) and the protein expression level (NH). Furthermore, and also similar to human NP samples in which TNF-α is known to be continuously expressed at basal levels (93), TNF-α could also be detected in adjacent discs (NA) suggesting to be a possible trigger of an early stage inflammation for IVDH.

Histological investigations demonstrated that most herniated IVDs exhibited an epidural inflammatory response, ranging from acute invasion of neutrophils to formation of chronic granulation tissue. Histological analysis of underlying inflammatory pathways demonstrated activation of the MAPKs ERK and p38 in most samples, whereas pJNK was rarely detected. MAPKs are signal transduction pathways that are activated by a multitude of stimuli, ranging from environmental, mechanical and osmotic stress to growth factors, cytokines and reactive oxygen species, with ERK and p38 controlling inflammation, catabolism as well as cell growth, differentiation and viability/death (27). The fact that the MAPKs pathways are shared between the cytokines might explain the correlation on the protein level in herniated samples of IL-1β with IL-6, IL-8, and TNF-α (94).

In accordance with our results, *in vitro* cell culture studies and rodent *in vivo* studies have previously demonstrated that p38 and ERK are activated in inflammatory environments (95–100), controlling a variety of metabolic functions associated with disc pathology, including proteoglycan degradation (96, 97). Furthermore, the decrease in osmotic pressure observed during herniation in our canine patients may also play a causative role in MAPK activation as previously demonstrated for bovine IVDs and the ERK pathway (101). Importantly, activation of p38 and ERK in our canine samples may not only induce downstream expression of additional inflammatory mediators and matrix degrading enzymes and hence play an important role in disease progression [reviewed in Ref. (27)], but may furthermore be a means to initiate resorption of non-contained herniated disc tissue (102–104).

Despite providing interesting new findings on the inflammation in canine IVDH, the present study has several limitations that should be considered. The dog size as well as the morphologic size of the herniated disc tissue imposed limitations on the number of performed tests. The quantity of surgically excised canine disc material is smaller than specimens collected in humans. Therefore, we could not divide all samples equally for PCR and ELISA testing as planned in the study design, resulting in variations in group size. Due to the non-predictable onset of canine IVDH, the group could neither be age nor gender matched, possibly influencing the results. Furthermore, most of our patients were presented at a late stage of the disease, which may explain why we might have missed the peak expression of some cytokines in the acute stage of the disease.

## Conclusion

Inflammation in the epidural compartment plays a central role in the pathophysiology of canine IVD herniation and significantly influences the course and outcome of the disease. Working with a clinical canine model not only provides the possibility to overcome animal experimentation, but furthermore allows (different from human studies) the analysis of adjacent, NA discs. Although expression of key cytokines found in human IVDs could also be demonstrated in canine tissue, the inflammatory mechanisms accompanying IVDH in dogs partially diverge from humans. These differences need to be considered when using dogs as a model for human medicine. In dogs, IL-6 seems to play an important pathological role, but further investigations on IL-6 as a potential therapeutic target in canine patients will be needed, especially when considering its likely ambivalent role.

## Ethics Statement

This study was carried out in accordance with the recommendations of “Animal Research Committee: Tierversuchsbewilligung BE 14/12, Switzerland.” The protocol was approved by the “Animal Research Committee: Tierversuchsbewilligung BE 14/12, Switzerland.” Furthermore, all patient-owners signed an owner consent form.

## Author Contributions

MM contributed to the conception and design, acquisition and data, analysis and interpretation of data, drafting of the manuscript, critical revision of the manuscript for important intellectual content, and statistical analysis. SF contributed to the analysis and interpretation of data, drafting of the manuscript, critical revision of the manuscript for important intellectual content, and the statistical analysis. DS contributed to the conception and design, critical revision of the manuscript and intellectual content, funding, and final approval of the version to be published. AK contributed to acquisition and data, interpretation and analysis of data, critical revision and administrative, technical, or material support. FF and KW-K contributed equally to the conception and design, analysis and interpretation of data, drafting of the manuscript, critical revision of the manuscript for important intellectual content, obtaining funding, administrative, technical or material support, supervision, and final approval of the version to be published. All authors agreed to be accountable for all aspects of the work in ensuring that questions related to the accuracy or integrity of any part of the work are appropriately investigated and resolved. We thank Prof. Dr. Michael Stoffel (Vetsuisse Bern, Veterinary Anatomy) for his support with histology.

## Conflict of Interest Statement

KW-K (08/03/2017) Relationships Pertaining to Submitted Manuscript—Money payed to the institution: 37.500 $ (CABMM Start-up Grant) Relevant financial activities outside the submitted work—Consulting Money payed to you: 85.000 $ (Schön Clinic Group). All other authors declare that the research was conducted in the absence of any commercial or financial relationships that could be construed as a potential conflict of interest.
